# Assessing the Influence of Compost and Biochar Amendments on the Mobility and Uptake of Heavy Metals by Green Leafy Vegetables

**DOI:** 10.3390/ijerph17217861

**Published:** 2020-10-27

**Authors:** Agnieszka Medyńska-Juraszek, Magdalena Bednik, Piotr Chohura

**Affiliations:** 1 Institute of Soil Sciences and Environmental Protection, Wroclaw University of Environmental and Life Sciences, Grunwaldzka 53 St., 50-357 Wroclaw, Poland; magdalena.bednik@upwr.edu.pl; 2 Department of Horticulture, Wroclaw University of Environmental and Life Sciences, Grunwaldzki Sq. 24a, 50-357 Wroclaw, Poland; piotr.chohura@upwr.edu.pl

**Keywords:** biochar, compost, amendments, heavy metal, uptake, vegetables, contamination

## Abstract

Municipal green-waste compost and wheat straw biochar amendments were assessed for their assistance in regulating the mobility of Cu, Pb, Zn, Cd, Cr and Ni and the uptake of these metals by five commonly grown green leafy vegetables (radish, lettuce, dill, spinach and parsley). The amendments were applied alone or combination of both in 5% and 10% (*v*/*w*) doses to soil contaminated with heavy metals. Vegetables were grown for eight weeks under greenhouse conditions, and in collected samples plant uptake and metal speciation in soil after sequential extraction procedure (BCR) were analyzed by Microwave Plasma Atomic Emission Spectrometer (MP-AES). The results of our study show that organic amendments noticeably reduced the uptake of heavy metals by various leafy vegetables, with the best result of reduced leaf accumulation for single biochar and biochar–compost mix application at higher dose. Single application of green-waste municipal compost may have adverse effects on heavy metal uptake, increasing the risk of vegetable contamination with Zn, Pb and Cr. This study recommends careful selection of vegetables for cultivation when organic fertilizers are applied to soil with elevated contents of trace elements or co-application of compost in mix with biochar to mitigate possible negative effects and human health risk.

## 1. Introduction

Heavy metal contaminated soil has become a global concern affecting world food production [1,2,3]. Excessive accumulation of trace elements in agricultural soils leads to elevated metal uptake by crops and thus affects food quality and safety [4,5,6]. Vegetables are important edible crops, being an essential part of the human diet. In many countries, vegetables are exposed to elevated concentration of metal(oids) by various means, mainly industrial emission, sewage sludge application and fertilization of soil. Moreover, heavy metals can be accumulated at high levels in the edible parts of plants, even when present in soil at low concentrations [7]. Thus, vegetable consumption may cause adverse effects instead of bringing benefits to our health. Trace elements contamination in soil is a challenging issue of risk management, as heavy metals do not degrade with time and therefore remain persistent in the environment [8]. In addition, conventional methods of remediation are usually costly, long-lasting and not necessarily effective [9]. An interesting tool for reducing the risk related to heavy metal presence in soil, especially on soil used for food production, is the use of organic amendments such as compost or biochar. With the rapid development of urbanization and a large quantity of municipal solid wastes (MSW) generated each year, composting has become an effective management of the MSW for recycling and converting organic waste into a useful product [10,11]. Compost is a high quality organic fertilizer which can replenish various nutrients, increase microbial activity and improve soil physical and chemical properties [12]. With increasing interest of EU citizens for organic farming and home-garden vegetable cultivation, green-waste compost has become one of the most popular growing substrates. Nonetheless, the application of compost can lead to the accumulation of heavy metals in soils and plants, and the entrance of metals into the food chain or groundwater can threaten human health and the environment [3,13,14,15]. A complementary material that can be obtained from various sources of biomass is biochar. Apart from agricultural purposes, the pyrogenic conversion of biomass into persistent charcoal products, commonly referred to as biochar, has been proposed as an effective means to increase terrestrial carbon storage, thus mitigating global warming caused by anthropogenic greenhouse gas emissions [16]. In general, biochars have high pH, contain organic functional groups [17] and have a microporous structure and a large surface area [18]. Those properties enhance sorption capacity, potentially making biochar an effective sorbent for pollutants [19], and increase soil reaction, which also reduces heavy metal mobility, as the majority of metals are less mobile in soil with higher pH values [20]. To reinforce the effect of this two soil amendments, compost and biochar can be mixed thoroughly to improve each other’s properties [21]. The combination of compost and biochar to restore soil has been researched recently, showing higher efficiency for reducing soil heavy metal toxicity when both components were mixed together [22,23]. We hypothesized that biochar addition can reduce bioavailability of heavy metals in compost, reducing the risk of trace element migration to plant tissues. Numerous studies on remediation by compost or biochar focus on certain ratio of amendments, while studies including different ratios of both substrates are rare. In addition, the effects of metal speciation or heavy metal uptake by different plant species are not thoroughly described. Therefore, in our study, we investigated the effect of the biochar, compost and biochar-compost combinations with different ratios on the mobility and availability of heavy metals. The main objectives were: (I) to evaluate changes of soil sorption properties amended with various biochar, compost and biochar–compost mixtures; (II) to analyze the effects of amendments on Cu, Zn, Cd, Cr, Pb and Ni speciation, mobility and bioavailability in multi-contaminated soil; and (III) to assess the efficacy of tested amendments and mixtures as immobilizing agents in order to reduce heavy metal uptake by green leafy vegetables grown on soils with elevated concentrations of contaminants. 

## 2. Materials and Methods 

### 2.1. Material Properties and Heavy Metal Content—Before the Experiment

#### 2.1.1. Substrate Collection (Soil, Biochar and Compost)

Sandy soil contaminated with heavy metals was collected from the top 30-cm layer of a copper smelter area (Poland) (51°41′18.7″ N 16°01′46.8″ E) and classified as Fluvic Brunic Arenosol according to Food and Agriculture Organization of the United Nations, World Reference Base, FAO WRB [24]. Heavy metal emissions from the smelter have been significantly reduced during the last decades; however, in the surface soil layer, the concentrations of metals are still elevated [25]. Compost used for experiment was produced in Wroclaw Biological Treatment of Municipal Wastes Plant (Wroclaw, Poland). The substrate was biodegradable green wastes from urban parks and gardens, composted for 12 weeks in prisms. The plant has an appropriate permit required by Polish law, regulating the conditions of composting organic wastes. Biochar used for experiment was derived from wheat straw (*Triticum* L.) in Swidnica Industrial Equipment Factory, Poland. Standard properties of soil, biochar and compost before use in the experiment were determined at Wroclaw University of Environmental and Life Sciences (WUELS) (Table 1). 

#### 2.1.2. Analyses of the Substrates

Analyses for characterization of substrates (soil, biochar and compost) were conducted on air dried materials as follows. The pH in water was measured in 1:5 (*v*/*v*) ratio using pH-meter (Mettler-Toledo, Graifensee, Switzerland). Cation exchange capacity, determined as the sum of base cations, was measured on an MP-AES 4200 Spectrometer (Agilent Technologies, Santa Clara, CA, USA) at pH 7.0 after extraction with 1 M ammonium acetate. Total organic carbon content in soil and compost was measured using CS-MAT 5500 analyzer (Ströhlein, Kaarst, Germany, currently Bruker AXS Inc., Madison, WI, USA). Total nitrogen content was determined by Kjeldahl method, on N analyzer (Buchi Labortechnik GmbH, Essen, Germany). Elemental composition of biochar (C, N) was determined on elemental analyzer (CE Instruments Ltd., Hindley, UK). The specific surface area of biochar (SSA) was measured using a TrisStar II 3020 (Micrometrics^®^, Norcross, GA, USA) surface area analyzer (N2-BET method) [18]. Content of ash in biochar was measured by dry combustion at the temperature 550 ℃. The soil texture was determined after dispersion with hexametaphosphate-bicarbonate, with hydrometer method for silt and clay fraction and sieved for sand [26]. Properties of substrates used for the experiment (soil, biochar and compost) are summarized in Table 1.

Content of heavy metals in substrates was measured as a semi-total content after microwave digestion with 10 mL HNO_3_, by EPA 3051A method [14]. Measurements were performed on MP-AES 4200 (Agilent Technologies, Santa Clara, CA, USA). Metal and metalloids results are provided as averages from triplicate experiments with the relative standard deviation (RSD), calculated by MP Expert Software Agilent Technologies. The maximum relative standard deviation (RSD) between replicates was set to 5%. Values that were above 5% were not included in the statistical analyses. To avoid analytical errors, standard solutions (from LGC Standards Ltd., UK) for MP-AES 4200 were used for calibration and certified reference materials as follows: RTH 953 Heavy Clay Soil from LGC Promochem (LGC Standards Ltd., Teddington, UK) and CRM055 (Honeywell Fluka, Charlotte, NC, USA) were analyzed with every sample set. The recovery of metals and arsenic from Certified Reference Material (CRM) was 82–96% and the maximum values of RSD were 3.4%. 

The obtained results of heavy metal and metalloid content were compared with the local soil and biochar quality standards, assuming agricultural use [27]. Exceeded concentrations of Cu, Pb and Cd indicate soil contamination. Biochar quality is not legally regulated in the research area; however, the substrate fulfills proposed international standards regarding the possibility of application in soil [28]. In the case of compost, there are no specific regulations regarding the contamination of the substrate. Heavy metals and metalloid contents are presented in Table 2.

### 2.2. Design of the Experiment

The experiment was set up in rectangular plastic pots (60 cm × 16.5 cm × 14 cm), approximately 4 L volume. Experimental trials are summarized in Table 3. 

**Figure 1:**
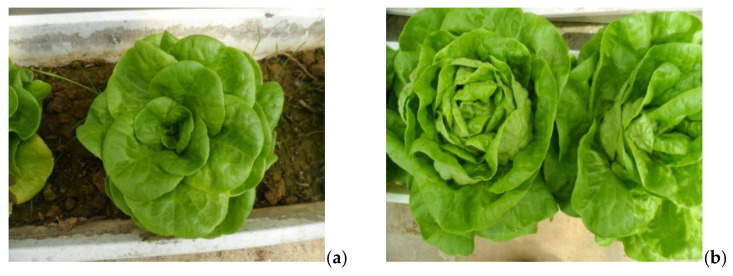
Lettuce growth in fifth week of the experiment: (**a**) on control soil without compost/biochar amendments (SC); and (**b**) on soil with 5% biochar + 5% compost (5BC + 5C).

Organic materials were mixed with the entire volume of soil, as in the pots it is difficult to divide material into tillage and non-tillage layer. Vegetables commonly grown by gardeners (radish, *Raphanus sativus* L. *var. sativus*; spinach, *Spinacia oleracea* L.; parsley, *Petroselinum crispum* (Mill.) Fuss.; dill, *Anethum graveolens* L.; and lettuce, *Lactuca sativa* L.) were sieved on prepared soil. Each variant was set up in two replicates. The pots were placed in a greenhouse and watered with distilled water as needed. Plants were grown in controlled light and temperature conditions for eight weeks, until the edible parts were fully developed (Figure 1). 

### 2.3. Chemical and Physical Analyses of Soil—After the Experiment

After eight weeks of incubation, the soil was collected from pots to repeat the analyses of standard chemical properties. The material was air-dried, homogenized, passed through a 2-mm sieve and then stored in dry place at room temperature. Analyses of pH, CEC, TOC and TN were repeated by the same methods as described in Section 2.1.

Soil physical properties (bulk density and water holding capacity) were determined using Kopecky cylinders of the volume 100 cm^3^. Water capillary capacity and field capacity were measured on sandy block (pF 0–2.0). Retention at higher pF values were determined with the sand/kaolin block and Richard’s apparatus (Eijkelkamp Soil & Water, Giesbeek, The Netherlands) [29]. 

### 2.4. Heavy Metals Content 

#### 2.4.1. Analyses of Heavy Metal Content in Soil

BCR sequential extraction procedure was performed to determine heavy metal speciation in soil after application of tested organic amendments. This procedure can be described briefly as follows: Step 1 (Exchangeable and weak acid soluble fraction): a 1-g soil sample was extracted with 40 mL of 0.11 mol L^−1^ acetic acid solution by shaking for 16 h. Step 2 (Reducible fraction): 40 mL of 0.5 mol L^−1^ hydroxylammonium chloride solution were added to the residue from Step 1, and the mixture was shaken for 16 h. Step 3 (Oxidisable fraction): 10 mL of 8.8 mol L^−1^ hydrogen peroxide solution were carefully added to the residue from Step 2. The mixture was digested for 2 h at 85 °C in presence of 10 mL of H_2_O_2_. The residue was extracted with 50 mL of 1 mol L^−1^ ammonium acetate solution, adjusted to pH 2.0 and shaken for 16 h. The extract was separated, and the residue was washed as in previous steps. Residue from Step 3 (Residual fraction): the residue from Step 3 was digested with aqua regia, to keep the same volume/mass ratio: 7.0 mL of HCl (37%) and 2.3 mL of HNO_3_ (70%) were added. For more detailed description, see the work by Pueyo et al. [30]. The obtained extracts representing four fractions were analyzed to determine heavy metal content on MP-AES 4200 (Agilent Technologies, Santa Clara, CA, USA). 

#### 2.4.2. Analyses of Heavy Metal Content in Plant Tissues

To determine content of heavy metals in plants, leaves and roots (for radish) were collected from the treatments at the end of experiment and washed in distilled water to reduce the risk of external contamination of the material with soil and dust. Then, samples were dried at 55 ℃ for 12 h in a laboratory dryer (POL-EKO, Wodzisław Śląski, Poland). The content of heavy metals in plant tissues was determined with MP-AES 4200 (Agilent Technologies, Santa Clara, CA, USA) after microwave digestion with mixture of 36% H_2_O_2_ and 10 mL of concentrated HNO_3_, following US-EPA (United States Environmental Protection Agency) 3052A method on STAR D digestion system (Milestone, Shelton, CT, USA).

To investigate the ability of plants to accumulate heavy metals in the tissues in relation to metal content in the soil, bioaccumulation coefficient (BAC) was calculated. This indicator evaluates the effectiveness of metal accumulation and is used mainly for predicting phytoremediation efficiency by different plant species. However, we assumed that BAC can also be used to estimate the potential for trace element accumulation in edible plants grown on soil with elevated concentrations of heavy metals, which might be helpful in health risk predictions. BAC of toxic metals in leaves was estimated by calculating the ratio of total metal content in plant leaves to that of total metal content in the soils: BAC = (Metal) leaf/(Metal) soil [31,32]. 

### 2.5. Data Analysis

The obtained data of soil chemical properties were statistically analyzed using Past 3.25 Software (Oslo, Norway). To determine significant differences, analysis of variance was undertaken at *p* = 0.05. The significant effects between treatments were detected using Tukey’s pairwise at significance level *p* = 0.05. Graphs were prepared with GraphPad Prism version 8.0.1 for Windows, GraphPad Software (San Diego, CA, USA).

## 3. Results

### 3.1. Effect of Amendments on Soil Physical and Chemical Properties 

After eight weeks of growth, noticeable effects of organic amendments on soil chemical properties were observed. Each of the amendments increased soil pH, but it was not always statistically significant. Addition of 5% *v*/*w* of biochar (5BC) increased soil pH by 0.58 unit. A double dose of biochar (10BC) had similar effect; however, in both treatments, there were no significant differences compared to SC (*p* > 0.05). A significant pH increase was observed in soil amended with compost in variant 10C (*p* = 0.013). Small doses of compost (5C) had no significant effect on soil pH. The combination of biochar and compost showed more positive effect at higher doses (10% *v*/*w* in 10BC + 10C) and soil pH increased significantly (*p* = 0.015) by 1.43 unit, compared with SC treatment. Similar results were obtained for cation exchange capacity and significant increase was observed in 10C (*p* = 0.03) treatment and combination 10BC + 10C (*p* = 0.02), while no effect on CEC was observed in BC-treated soil.

**Figure 2:**
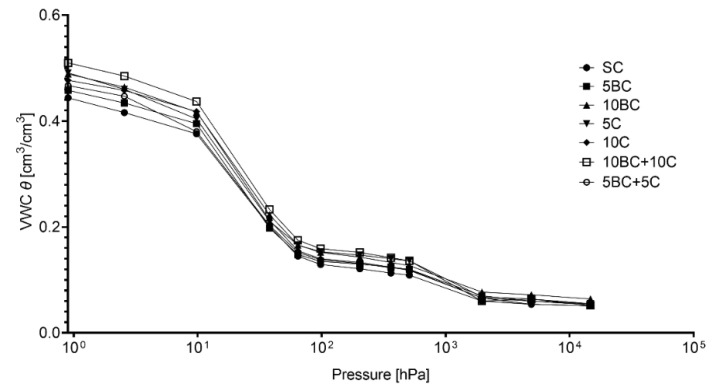
Water retention curves for experimental treatments. VWC, volumetric water content. Abbreviations of the treatments are defined in Table 3.

As expected, organic amendments increased total organic carbon content, proportionally to the dose of material and input of organic matter (Table 4). High amounts of the amendments resulted in statistically significant differences in TOC, compared with control soil. The best result was obtained when compost and biochar were applied in 10% dose or in combination. Application of BC and BC in mix with compost caused amplification of C:N ratio from 12:1 to 32:1, confirming that nitrogen deficiency may be higher when carbon rich materials are applied to soil. Tested organic amendments improved water content and water holding capacity of sandy soil (Figure 2). The lowest volumetric water content (VWC) was determined in SC treatment (maximum water content: 0.44 cm^3^/cm^3^) increasing up to 0.51 cm^3^/cm^3^ in 10BC + 10C soil. Water available for plants increased by 18% in 10C, by 17% in 10BC and by 24% in 10BC + 10C, compared with SC. 

### 3.2. Sequential Extraction of Heavy Metals

**Figure 3:**
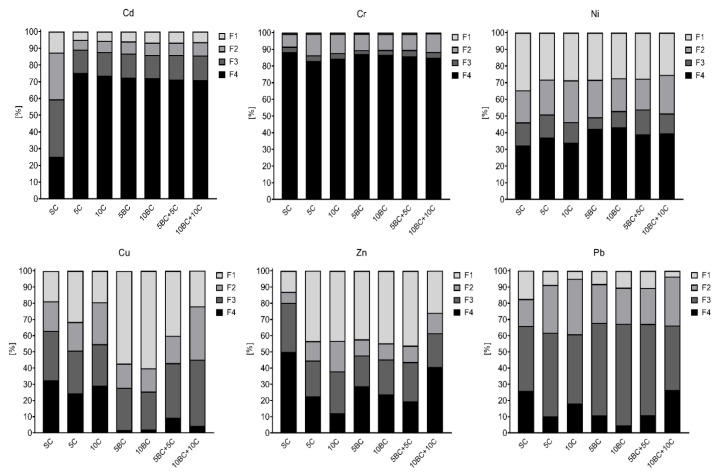
Speciation of Cu, Zn, Pb, Cd, Cr and Ni in amended soil—share of fractions in total metal content (100%). F1, easily soluble fraction; F2, reducible, bound to Fe and Mn oxides; F3, oxidizable, bound to organic matter; F4, residual fraction. Abbreviations of the treatments are defined in Table 3.

The effect on metal speciation in tested soil depended on the type of the amendment (compost, biochar and mix of both) and the ratio. For copper, an increase of exchangeable forms was observed after addition of both organic amendments, however the change was more significant (*p* > 0.05) compared to compost treated soil. Application of biochar caused increase of Cu in fraction F1 from 18.7% in SC to 57.2% in 5BC and 60.1% in 10BC (Figure 3).

Co-application of both BC and C caused a shift of copper species from residual fraction F4 to fraction F3 (bound with organic matter, oxidizable) in 10BC + 10C treatment; however, in 5BC + 5C, the element remained in not easily available form. A similar effect was observed for Zn, and both BC and C caused a significant increase (*p* > 0.05) of Zn in easily exchangeable forms compared to SC. Nonetheless, 10BC + 10C showed very good results and most of the Zn was shifted to not bioavailable forms in the tested soil. The most visible change was observed for Pb species after addition of both organic amendments. Application of organic materials reduced the bioavailability of Pb in tested soil, shifting the metal from exchangeable fraction F1 to fraction F3 (bound with organic matter), respectively. Application of organic material caused a shift of Pb from residual fraction F4 to fraction F3 (bound with organic matter), improving the important role in Pb binding with organic matter. The best effect was achieved in 10BC + C, where only 3.6% of Pb compared with 17.5% in SC was in readily available fraction F1. The most noticeable metal immobilization after addition of both organic amendments was observed for cadmium. Almost 75% of Cd was in residual fraction F4 when compost was applied, compared with 25% in fraction F4 for SC. Biochar, compost and combination of BC and C had similar beneficial impacts on Cd immobilization. No significant effect of tested amendments was observed for Cr and Ni. Almost 90% of chromium in tested soil was in residual forms. For nickel, a slight reduction of exchangeable forms was observed after organic materials application, the highest being for 10BC + 10C variant. In general, the efficiency of metal immobilization, if noticed in a given experimental set, was higher with the increase of amendment dose from 5% to 10%. 

### 3.3. Heavy Metals Accumulation in Plants

**Figure 4:**
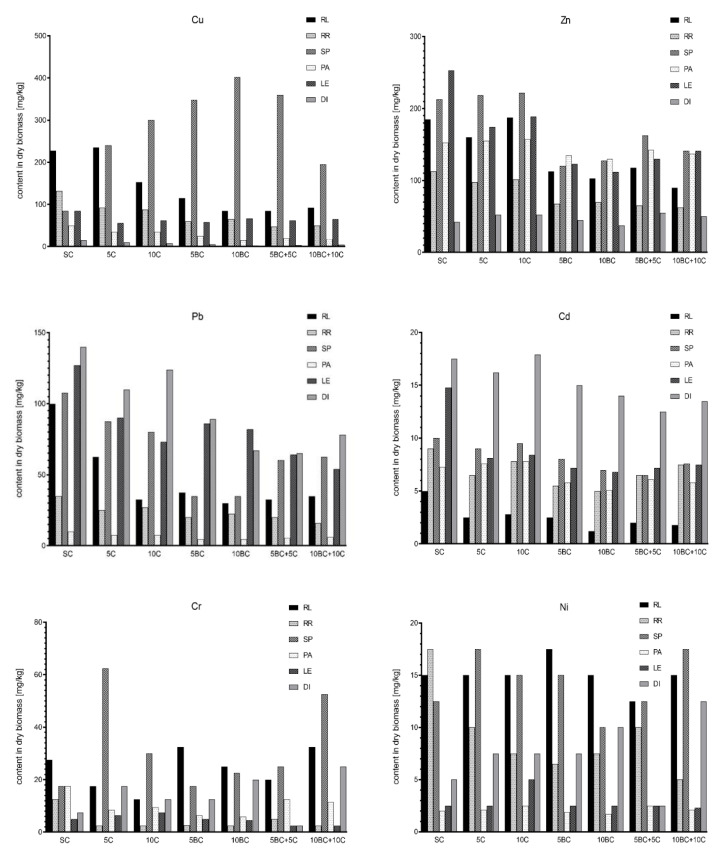
Content of Cu, Zn, Pb, Cd, Cr and Ni in vegetables grown on soil amended with biochar, compost and their combination. RL, radish leaf; RR, radish root; SP, spinach; PA, parsley; LE, lettuce; DI, dill. Abbreviations of the treatments are defined in Table 3.

The response to organic amendments and effects on heavy metal content in plant tissues varied among plant species, organic amendments and metal type. In general, the best effect of reduced metal content in plant tissues was observed for treatments with biochar and combination of biochar and compost; however, the effect also depended on the vegetable species (Figure 4). The highest content of Cu, Zn, Cr and Ni was determined in spinach leaves, while Pb and Cd were the most abundant in dill leaves. Parsley, in turn, was the vegetable accumulating the lowest amounts of tested metals. Application of organic amendments showed some adverse effects on heavy metal uptake depending on the material (compost or biochar). For spinach and dill, application of compost alone increased the accumulation of Cu, Pb, Cd and Cr, while application of biochar often reduced the content of trace elements in tissues. In most cases, application of compost and biochar in combination reduced the negative effect caused by compost, decreasing the content of heavy metals in tested plant species. In radish leaves, BC + C combination decreased the amount of Cu and Zn, respectively, by 63% and 51%, while, in radish roots, it was reduced by 50%, compared with contents observed in SC. Similar good effects were noticed for lettuce, and biochar application allowed reducing Zn presence in plant tissues by 51% in 5BC and by 57% in 10BC treatments. Application of compost and biochar, especially in combination, reduced the content of Pb and Cd in almost all tested plant species. In 10BC + 10C treatment, the presence of Pb in spinach was reduced by 42%, in dill leaves by 44% and in lettuce by 57%. In turn, the content of Cd in plant tissues was 50% lower than in control set for lettuce and about 25% lower for the other edible parts of the examined plants. However, some negative effects of organic amendments were also observed. Application of compost enhanced the accumulation of Cr by spinach and dill, respectively, by 357% and 233% on 5C treatment. The worst results were observed for Ni, as compost, biochar and combination of both increased significantly (*p* > 0.05) the uptake of this metal by all tested vegetable species.

BAC values depended strongly on the plant species and type of metal. For all tested vegetables, the accumulation of most metals (Cu, Zn, Pb, Cr and Ni) was the highest in spinach and the lowest in lettuce. BAC for Cu and Cd was ≤1, suggesting that bioaccumulation of this elements in tested plant species was usually very low, regardless of heavy metal content and speciation. Zinc, in turn, strongly accumulated in tested plants, especially in radish, spinach, parsley and lettuce (BAC > 5). The second most frequently accumulated metal in vegetables was lead, with the highest BAC values for dill leaves (2.81). For Cr and Ni, BAC values higher than 1 were estimated for radish, spinach and dill, suggesting that these plants are able to accumulate more of this trace elements compared with lettuce or parsley grown under similar conditions. In general, application of organic amendments, especially biochar and combination of both materials (biochar and compost) in high rates (10%), decreased BAC of tested metals in comparison with control set (Table 5). 

## 4. Discussion

One of the expected effects after incorporation of organic amendments is the improvement of soil water content. In general, we confirmed that both tested organic agents and their combination slightly enhance water properties of light textured soils. Biochar is able to hold water on the surface of its fine, porous particles, as well as increases the content of mesopores in soil [33,34]. However, obtained results were less noticeable than often described in the literature (e.g., [35]). Different effects of biochar may arise from its properties (feedstock and porosity), parameters of pyrolysis (temperature and duration), soil texture and the dose and application method [36,37]. It is stated that hydrophilic material should be produced at 300–500 °C [35]. Biochar in the experiment was obtained at 550℃, therefore its hydrophilicity is unclear. Some authors have also claimed that shredding the biochar may destroy its porosity and reduce water binding [38]. 

The most desired effect of organic amendment application is reduced content of trace elements in edible parts of plants, which are not considered as plant micronutrients; however, their presence in vegetables intended for the consumption have potential adverse effects on human health [7]. The presented results confirm that biochar and biochar in mix with compost can have profound effects on heavy metal uptake by different plant species, especially when applied in higher dose (10% BC and 10% C). The novel finding of our research is that changes of heavy metal mobility are related to the properties of materials and less to the changes of soil properties occurring after amendments application. The results of the study demonstrate that application of different organic materials and combinations of both can affect sorption effectiveness, however this effect varied with different metals, different ratios of materials applied to soil and properties of biochar and compost, as both components could contribute the sorption process in different ways. In general, biochar application refills soil organic matter with very stable carbon forms [39]. Biochar, due to the occurrence of oxygen functional groups [40] on its surface, could support heavy metal retention. Compost as a source of dissolved organic carbon (DOC) plays the most important role in increasing soil sorption capacity and heavy metal retention, which was also observed by Beesley et al. [41]. The results of our study show that compost can be an important donor of unstable form of organic matter and exchangeable forms of trace elements. This fact should be considered when compost application is recommended as organic fertilizer [42,43]. Combining biochar and compost was beneficial, as properties of both materials can be enhanced, and the potential drawbacks are minimized. However, both organic materials as well as their combination may also have adverse effects depending on the trace element and basic speciation of heavy metal in soil before amendment application [44]. The most beneficial effects after incorporation of tested amendments were observed for Pb and Cd immobilization. Retention of both elements in contaminated soil was significantly improved when 10% mix of biochar and compost was applied to soil. After compost and biochar–compost mix application, cadmium speciation increased by 50% in residual forms that are not bioavailable for plants. The results are in agreement with previous studies [45]. Opposite effects were observed for Cu and Zn, as higher amounts of these trace elements were determined in exchangeable and easily soluble forms after biochar, compost and mix application to tested soil. The findings of our study are in agreement with our previous observations [18] and with the results of other authors, describing that application of organic matter, especially compost, increases solubility of Zn [18] and Cu [22]. For example, Beesley et al. [22] found that, after contaminated soil was treated with both biochar and green-waste compost, the concentrations of Cd decreased significantly, while the concentrations of labile Cu increased by more than 30 times. Tripti et al. [4] reported that Cu smelter influenced soil, increasing the content of more labile Cu, which can be readily taken up by the plants. Karami et al. [17] explained in an experiment with compost and biochar that large reductions in Pb solubility in soil, compared to Cu, may be related to humified complexes. Tang et al. [46] confirmed this thesis, performing a similar experiment with biochar and compost mixture as amendment to contaminated soil. They showed that extractable Cu content increased when compost was added to soil due to the presence of humic acids dissolving and complexing copper at lower pH. Cr and Ni are less studied metals and knowledge about potential effects on the mobility of both elements after biochar and compost application to soil is scarce. The result of our study do not show any significant changes of Cr and Ni availability in tested soil, probably due to very low concentrations of metals in the contaminated soil and the higher concentration of chromium and nickel in compost, contributing to the pool of available forms of these metals in the amended soil. Green leafy vegetables are a popular food choice and an important component of the diet for most humans worldwide. With increasing trends for gardening and organic vegetable growing by UE citizens, more attention should be paid to education and food quality assurance to garden owners [47,48]. In most cases, it is believed that growing vegetables without mineral fertilizers and pesticides is safe. Therefore, soil testing is considered unnecessary when organic gardening is performed. As the heavy metal uptake from soil to plants is a function of the physical and chemical nature of the soil or growing medium and is altered by innumerable environmental and human factors, heavy metal contamination of soils is one of the greatest concerns in Europe [49]. Plants adsorb a number of elements from soil. Some of them have biological functions, but toxic metals, if present in soil solution, can be also easily taken up by plants and transferred to higher organisms in the food chain. Knowledge about the behavior of particular trace elements in soil and factors influencing their bioavailability and uptake by particular edible plant species is very important. Plants take up essential and non-essential elements from soils in response to concentration gradients induced by selective uptake of ions by roots or by diffusion of elements in the soil [50]. As plants can only uptake heavy metals dissolved in soil solution, application of organic amendments should immobilize metals in solid phase [51,52]. Our results show the opposite effect of both organic amendments (biochar and compost) and the synergic effect of biochar and compost combination on bioavailability of metals in soil or their uptake by tested plant species. Radish, spinach, dill, parsley and lettuce are popular vegetable choices in the diet of Europeans, as they can be easily grown in gardens, representing species with different abilities for particular heavy metal uptake, as previously described by other authors [53,54,55,56]. Different vegetable crops grown on heavy metal contaminated soil showed marked difference in metal accumulation, uptake and distribution pattern depending on plant species, growth stages, types of soil and metals, soil conditions or weather. Crop species also showed remarkable difference in metal concentration of various plant parts or they can vary between different varieties in one species [57]. This makes it more difficult to predict which cultivated vegetable species on soils with elevated contents of toxic elements will bring more adverse effects to human health. The results of our study show that tested vegetable species have similar ability for zinc uptake. As an important micronutrient, plants do not develop barrier mechanisms for its uptake and in general zinc is easily transferred in plant and stored in older leaves [57]. The metal taken up by vegetables in the lowest amount was Cu, which can be explained by the low solubility of this element in tested soil. Copper is mostly accumulated in roots and storage cells [58], and, as only aboveground biomass was tested, the difference between tested species could be less noticeable. The higher concern is about lead and cadmium, as elements with high toxicity. In general, BAC values estimated for tested species showed that cadmium is not accumulating (BAC < 1) in all tested leafy vegetables. Plant roots act as barriers in metal translocation from soil to plant, which is a well described phenomenon for Cd [6,59,60], which explains the low accumulation of element in tested green leafy vegetables. Pb accumulation was indicated for all tested species (BAC > 1) except radish leaves. The effect was strongly related with soil conditions, and higher values where obtained when vegetables were cultivated with single compost amendments. Similar findings were observed for cadmium in all compost treated soils. Increased BAC values for Pb and Cd cannot be explained by the increase of soluble forms of both metals in soil solution; however, the change of metal speciation (for Pb) or lower pH of compost amended soil (for Cd) may influence metal uptake under tested soil conditions [61]. Pb and Cd uptake by plants depends on several soil properties such as pH or soil organic matter content [58], which can explain the increase of BAC values when compost was applied to soil. Chromium is slightly available to plants and not easily translocated within plants, thus it is concentrated mainly in roots, as Cr^2+^ is bound to cell walls. Contents of Cr in plants are controlled mainly by the soluble Cr contents of the soil. Cr availability to plants is significantly limited [58]. The results of the study suggest that chromium can be accumulated in aboveground plant parts if present in soil in soluble forms [62]. For most of the tested plant species, except lettuce, BAC values were >1, indicating that application of organic amendments may increase the risk of Cr transfer to edible plant parts. Nickel as a plant nutrient is easily taken up by plants and transferred to leaves; however, this was not observed for all tested leafy vegetables, suggesting that the mechanism of Ni uptake depends not only on the bioavailability of the element in soil, but also on plant activity and varies between plant species [63]. The results of our study indicate that application of organic amendments as commonly used by gardeners (compost) or more advertised by researchers (biochar) can be helpful in mitigating the problem of metal uptake by edible plants; however, attention should be paid to the quality and initial content of toxic elements in substrate used as soil amendment. Application of single compost may increase the concentration of heavy metals in some vegetable species, bringing potential risk of adverse health effects. However, new solution of combining compost with biochar might be beneficial for toxic compounds mitigation in soil and reduction of metal transfer to edible plants. Both substrates have synergistic effect, becoming more efficient when applied together, as observed in the study. 

## 5. Conclusions

- The results of our study show that organic amendments noticeably reduced the uptake of heavy metals by various leafy vegetables, showing the best result of reduced accumulation for biochar variants and biochar combined with compost.
- Simple application of compost increased solubility of some trace elements initiating more intensive uptake and resulting in higher bioaccumulation of this contaminants in edible plant parts.
- Combining biochar and compost is recommended to enhance their beneficial properties and to minimize potential negative impact on environment.
- Spinach and dill were most capable of metal bioaccumulation. Therefore, it is recommended to avoid or reduce their consumption, if grown on potentially contaminated soil.

## Figures and Tables

**Table 1:** Properties of soil, biochar and compost used in the experiment.

Characteristic	Value
**Soil**	
Classification	Fluvic Brunic Arenosol (FAO WRB)
Texture	loamy sand (73% sand, 26% silt, 1% clay)
pH (in H_2_O)	4.87 ± 0.15 *
Total organic carbon (TOC, %)	0.97 ± 0.05
Total nitrogen (TN, %)	0.07 ± 0.02
Cation exchange capacity (CEC *, cmol/kg)	5.51 ± 0.13
**Biochar**	
Substrate	wheat straw
Pyrolysis time	30 sec.
Pyrolysis temperature	550 ℃
pH	9.86 ± 0.12
Total organic carbon (TOC, %)	55 ± 0.5
Total nitrogen (TN, %)	1.12 ± 0.03
Cation exchange capacity (CEC, cmol/kg)	63
Specific surface area (SSA, m^2^/g)	239 ± 4.5
Ash content (%)	32 ± 1.2
**Compost**	
Substrate	urban green waste
Composting time	12 weeks
Composting method	prisms
pH	5.91 ± 0.15
Total organic carbon (TOC, %)	12.2 ± 0.03
Total nitrogen (TN, %)	0.89 ± 0.02

* Values are means ± standard deviation (n = 3).

**Table 2:** Trace element content in substrates.

Metal (loid) (mg/kg)	Soil	Biochar	Compost
Cu	321 ± 3.5 * (100) ^a^	11 ± 1.1(70)	45.3 ± 1.3 *
Zn	32.4 ± 1.4 (300)	38 ± 0.4 (200)	22.0 ± 0.8 *
Pb	174 ± 2.1 (100) ^b^	2 ± 0.1 (45)	24.3 ± 1.1 (140)
Cd	6.2 ± 1.1 (2)	<0.01 (0.7)	1.9 ± 0.2 (5)
Cr	8.9 ± 0.6 (150)	3.5 ± 0.4 (70)	19 ± 1.4 (100)
Ni	9.2 ± 0.4 (100)	2.4 ± 0.5 (25)	7.0 ± 0.3(60)
As	1.3 ± 0.15 (10)	0.1 ± 0.0(13)	1.1 ± 0.2 *

* Values are means ± standard deviation (n = 3); ^a^ values in the brackets represent standard maximum values for soil and organic amendments according to Polish law regulation and recommendation for biochar; ^b^ The red color indicates over ranged metal contents in arable soils according to Polish soil standards.

**Table 3:** Summary of the treatments in greenhouse experiment.

Description	Abbreviation	Amendment Dose Equivalent (t/ha)
Control soil without organic amendments	SC	-
Soil + 5% (*v*/*w*) wheat straw biochar	5BC	42
Soil + 10% (*v*/*w*) wheat straw biochar	10BC	84
Soil + 5% (*v*/*w*) municipal green-waste compost	5C	42
Soil + 10% (*v*/*w*) municipal green-waste compost	10C	84
Soil + 5% (*v*/*w*) wheat straw biochar + 5% (*v*/*w*) municipal green-waste compost	5BC +5C	42 + 42
Soil+ 10% (*v*/*w*) wheat straw biochar + 10% (*v*/*w*) municipal green-waste compost	10BC + 10C	84 + 84

**Table 4:** Effect of biochar and compost on soil chemical properties after eight weeks of incubation.

Treatment	pH in H_2_O	CEC cmol (+)/kg	TOC %	TN %	C:N
**SC**	4.87 ± 0.03	5.58 ± 0.05	0.77 ± 0.05	0.03 ± 0.001	26:1
**5C**	5.65 ± 0.13 ns	6.57 ± 0.19 ns	0.97 ± 0.05 ns	0.08 ± 0.002 ns	12:1
**10C**	6.33 ± 0.17 *	11.12 ± 0.14 *	1.45 ± 0.16 *	0.12 ± 0.002 *	12:1
**5BC**	5.45 ± 0.14 ns	5.50 ± 0.08 ns	0.98 ± 0.09 ns	0.04 ± 0.002 ns	25:1
**10BC**	5.41 ± 0.12 ns	5.91 ± 0.18 ns	1.34 ± 0.05 *	0.07 ± 0.008 ns	19:1
**5BC + 5C**	5.71 ± 0.10 ns	7.47 ± 0.21 ns	1.91 ± 0.10 **	0.06 ± 0.005 ns	32:1
**10BC + 10C**	6.30 ± 0.13 *	9.54 ± 0.15 *	2.18 ± 0.02 ***	0.10 ± 0.002 *	22:1

Values are means with ± SD (n = 3). CEC, cation exchange capacity; TOC, total organic carbon; TN, total nitrogen; C:N, carbon to nitrogen ratio. Symbols indicate statistical significance between treatment and control set: ns, not significant (*p* > 0.05), * *p* ≤ 0.05, ** *p* ≤ 0.01, *** *p* ≤ 0.001. Abbreviations of the treatments are defined in Table 3.

**Table 5:** Bioaccumulation coefficient (BAC) of Cu, Zn, Cd, Pb, Cr and Ni in five species of green leafy vegetables grown on soil with different types and ratios of organic amendments (compost, biochar and mix of both materials.

Treatment	BAC						BAC					
	Cu	Zn	Cd	Pb	Cr	Ni	Cu	Zn	Cd	Pb	Cr	Ni
	Radish (Leaf)						Dill					
SC	0.71	5.75	0.80	0.57	3.09	1.63	0.05	1.32	0.80	2.81	0.84	0.54
5C	0.65	4.80	0.38	0.47	1.97	1.63	0.03	1.58	0.83	2.48	1.97	0.82
10C	0.44	4.80	0.41	0.29	1.40	1.63	0.02	1.34	1.09	2.62	1.40	0.82
5BC	0.36	4.12	0.38	0.39	3.65	1.90	0.02	1.65	0.94	2.28	1.40	0.82
10BC	0.29	3.51	0.17	0.30	2.81	1.63	0.01	1.28	0.66	2.03	2.25	1.09
5BC + 5C	0.29	3.90	0.29	0.27	2.25	1.36	0.01	1.83	0.54	1.80	0.28	0.27
10BC + 10C	0.40	2.56	0.26	0.25	3.65	1.63	0.02	1.42	0.56	1.97	2.81	1.36
	**Spinach**						**Lettuce**					
SC	0.26	6.60	0.62	1.61	1.97	1.36	0.26	7.86	0.73	2.38	0.56	0.27
5C	0.66	6.56	0.66	1.38	7.02	1.90	0.16	5.23	0.68	1.24	0.73	0.27
10C	0.86	5.69	0.70	1.39	3.37	1.63	0.18	4.84	0.64	1.23	0.84	0.54
5BC	1.08	4.40	0.37	1.21	1.97	1.63	0.18	4.51	0.91	1.09	0.56	0.27
10BC	1.39	4.37	0.35	1.02	2.53	1.09	0.23	3.84	0.81	0.99	0.51	0.27
5BC + 5C	1.21	5.40	0.50	0.94	2.81	1.36	0.21	4.32	0.53	1.04	0.28	0.27
10BC + 10C	0.83	4.02	0.45	1.11	5.90	1.90	0.28	4.02	0.39	1.09	0.28	0.25
	**Parsley**					
SC	0.16	4.74	0.06	1.17	1.97	0.22						
5C	0.10	4.65	0.06	1.16	0.96	0.23						
10C	0.10	4.03	0.07	1.14	1.07	0.27						
5BC	0.08	4.95	0.05	0.88	0.73	0.21						
10BC	0.05	4.45	0.04	0.74	0.66	0.18						
5BC + 5C	0.07	4.73	0.05	0.88	1.40	0.27						
10BC + 10C	0.08	3.90	0.04	0.85	1.29	0.23						

The color indicate the intensity of metal bioaccumulation: green, low bioaccumulation; yellow, medium; orange, high; red, very high bioaccumulation of given metal. Abbreviations of the treatments are defined in Table 3.
